# Fruit–Vegetable–Herb Blend‐Based Polyphenol‐Rich Food Powder: Physicochemical, Functional, Antioxidant, and Sensory Evaluation

**DOI:** 10.1155/ijfo/1373291

**Published:** 2025-09-28

**Authors:** Pramod K. Prabhakar, Sanket Yadav, Yogesh Kumar, Rajat Suhag, Giovanna Ferrentino

**Affiliations:** ^1^ Department of Food Science and Technology, National Institute of Food Technology Entrepreneurship and Management, Sonipat, Haryana, India, niftem.ac.in; ^2^ Department of Agricultural and Food Sciences, University of Bologna, Cesena, Italy, unibo.it; ^3^ Faculty of Agricultural, Environmental and Food Sciences, Free University of Bolzano, Bolzano, Italy, unibz.it

**Keywords:** freeze-drying, powder flowability, product development, solubility, thermal analysis

## Abstract

This study focuses on developing and characterizing polyphenol‐rich particulate systems from blends of guava, beetroot, orange, and mint juices in varying proportions. The resulting particles had high yields exceeding 90%. Physical properties showed bulk densities of 0.69–0.76 g/cm^3^ and good flowability as indicated by favorable Carr indices (CIs) and Hausner ratios (HRs). The powders demonstrated low moisture content (2.9%–3.15%) and water activity (0.35–0.36), alongside solubility (88.08%–89.74%), wettability (112.33–123.67 s), dispersibility (87.19%–93.50%), and hygroscopicity (30.65%–34.46%). Thermal properties such as diffusivity, conductivity, and volumetric heat capacity were recorded at 0.124–0.138 mm^2^/s, 0.13–0.14 W/mK, and 0.80–1.18 MJ/m^3^K, respectively. The powders retained substantial bioactive compounds, with total phenolic content ranging from 101.81 to 160.11 mg GAE/100 g and DPPH radical scavenging activity from 46.91% to 59.33%. FTIR analysis confirmed the presence of phenolics, proteins, and sugars. Sensory evaluation highlighted significant differences in consumer acceptability, with one formulation achieving top scores for appearance (8.3), texture (7.4), and overall acceptability (8.5). This study underscores the potential of fruit–vegetable–herb blends in creating functional, nutrient‐dense powders with broad applications and strong consumer appeal.

## 1. Introduction

Fruits and vegetables are essential components of a healthy diet, with their regular intake linked to a reduced risk of cardiovascular and coronary diseases, metabolic disorders, degenerative conditions, and certain cancers [1]. This health‐promoting effect is largely attributed to their abundance of fibers, vitamins, minerals, and bioactive compounds like polyphenols, flavonoids, carotenoids, and anthocyanins with potent antioxidant properties [2, 3]. Guava (*Psidium guajava* L.), often called as a “superfruit,” stands out for its rich content of vitamins A and C, alongside essential dietary minerals such as potassium and magnesium, all within a nutrient‐dense, low‐calorie profile. Its seeds further enhance its value with high levels of omega‐3 and omega‐6 polyunsaturated fatty acids and dietary fiber [4, 5]. Beetroot (*Beta vulgaris* L.) is characterized by its richness in betalains and phenolic compounds, which are linked to antioxidant, anti‐inflammatory, and immunomodulatory effects, as well as potential cancer‐preventive properties [6, 7]. Orange (*Citrus sinensis*), among the most widely used fruits for juice extraction, is celebrated for its abundance of antioxidants and well‐documented health benefits [8]. Mint (*Mentha spicata* L.), known for its medicinal qualities, supports digestive health, enhances cardiac function, and improves memory [9]. Additionally, its antimicrobial and antioxidant properties make it highly beneficial for overall health. With its rich menthol content, mint also plays a significant role in the food and beverage industry as a natural flavoring agent [10, 11].

Most fruits and vegetables are seasonal, with peak availability during specific times of the year. While fresh consumption is common during the harvest season, these fruits are often processed into products like juices, squashes, concentrates, jams, and pickles to ensure year‐round availability. The type of product is determined by the fruit’s composition. For instance, fruits with high sugar and pulp content are ideal for juice and squash production [12]. Although nutritionally valuable, some fruits and vegetables are challenging to utilize in product development because of their high acidity, bitterness, or astringency. For instance, it is recommended to dilute beetroot juice before consumption, as undiluted beetroot juice can cause burning sensations, dizziness, and diarrhea [13]. Blending two or more fruit and vegetable juices offers a practical solution for effectively utilizing them. This approach can enhance the nutritional value, aroma, and taste of the final product. Additionally, incorporating herbs such as mint not only adds an appetizing quality but also provides medicinal and therapeutic benefits.

Moreover, juices are highly susceptible to spoilage due to their high water and sugar content, which promotes microbial growth, including yeasts, molds, and bacteria. Enzymatic reactions further contribute to quality deterioration, limiting shelf life [14, 15]. To address these challenges, converting juices into a solid particulate system can be a convenient solution. Compared to liquid juices, solid particulate systems offer several advantages, including reduced volume and weight, easier packaging, improved preservation, handling, transportation, and storage, as well as an extended shelf life [16, 17]. Solid particles are commonly used as instant drink mixes, reconstituted with water to create refreshing beverages. Additionally, fruit powders serve as versatile ingredients in products such as baby food, desserts, fruit yogurts, soups, cakes, ice creams, and confectionery items [12, 18, 19].

This study presents the development of a novel composite particulate system derived from a unique blend of guava, beetroot, orange, and mint juices, formulated at varying concentrations. Unlike previous studies focusing on single‐source powders, this work integrates complementary fruit and herb matrices to synergistically enhance both functional and sensory attributes. The produced particles were extensively characterized for their physical (density, flowability, and morphology) and instant properties (solubility, wettability, dispersibility, and hygroscopicity), as well as their thermal behavior, including glass transition temperature, thermal conductivity, thermal diffusivity, and volumetric heat capacity. Moreover, a detailed assessment of antioxidant activities was conducted. To determine real‐world applicability, consumer acceptability was also evaluated. The original contribution of this study lies in its integrative approach to designing a functional, multijuice powder with enhanced stability, reconstitution behavior, and consumer appeal, advancing the development of health‐oriented, shelf‐stable powdered beverages.

## 2. Material and Methods

### 2.1. Materials

Fresh guava, beetroot, orange, and mint were obtained from a local market in Sonipat (India) and stored in cold storage for further use. Maltodextrin (dextrose equivalent ≤ 20), potassium sulfate (K_2_SO_4_), copper(II) sulfate (CuSO_4_), sulfuric acid (H_2_SO_4_), sodium hydroxide (NaOH), hydrochloric acid (HCl), boric acid (H_3_BO_3_), indicator methyl red, indicator bromocresol green, petroleum ether, phenolphthalein indicator, Folin–Ciocalteu (FC) reagent, anhydrous sodium carbonate, gallic acid, ethanol, 2,2‐diphenyl‐1‐picrylhydrazyl (DPPH), methanol, sodium nitrite, aluminum chloride, catechin, sodium sulfate, and ammonium nitrate were purchased from Sigma‐Aldrich. For the encapsulating agent, maltodextrin was chosen for its affordability, wide availability, high water solubility, low viscosity, and neutral flavor. Additionally, maltodextrin is effective in protecting flavor, color, and bioactive compounds under challenging environmental conditions [20, 21]. All solvents and reagents were of analytical grade.

### 2.2. Juice Extraction

**Figure 1:**
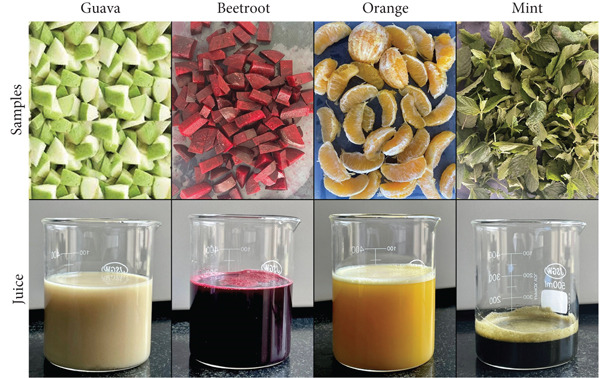
Cleaned and diced samples of guava, beetroot, orange, and mint, along with their extracted juices.

Guava, beetroot, orange, and mint were inspected to remove any signs of damage or spoilage and rinsed thoroughly. Before juice extraction, the fruits were diced as shown in Figure 1. Guava and beetroot were blended with water in a 2:1 ratio, while orange and mint were mashed without added water. The resulting pulpy mixtures were filtered through a muslin cloth to separate the juice from solids and fibers.

### 2.3. Formulations and Composite Particulate Preparation

The extracted juices were blended in varying proportions to create different formulations before freeze‐drying, as shown in Table 1. Guava was fixed at 50% in all samples due to its high vitamin C content (~173 mg/100 g) and strong base matrix properties [4]. The remaining 50% was systematically varied among beetroot, orange, and mint to investigate their combined effects on the nutritional, functional, and sensory properties of the product. Beetroot was included for its rich antioxidant profile, abundance of bioactive compounds, and natural pigments (betalains), contributing both health benefits and a visually appealing color. Orange, also a source of vitamin C (~53 mg/100 g), was incorporated to enhance the overall nutritional profile and add a complementary citrus note [20]. Mint was added in the lowest proportion (3%–6%) primarily for its aromatic contribution, aimed at improving the flavor profile without overpowering the blend. Prior to freezing, the total soluble solid (TSS) content of the juice mixtures was measured, ranging from 11.28% to 11.39%. To serve as an encapsulating agent, maltodextrin with a dextrose equivalent ≤ 20 was added to the prepared samples.

**Table 1:** Formulations of different composite particles.

**Sample**	**Guava (%)**	**Beetroot (%)**	**Orange (%)**	**Mint (%)**
S1	50	30	17	3
S2	50	27	19	4
S3	50	24	21	5
S4	50	21	23	6

A 5% portion of maltodextrin was incorporated into each juice blend, followed by homogenization using a high‐speed Ultra Turrax T25 homogenizer (Janke and Kunkel, Germany) at 9000 rpm for 8 min. The homogenized samples (150 mL) were placed into 500‐mL round‐bottomed flasks and frozen at −80°C for 12 h in a deep freezer. The freeze‐drying process was conducted using a Telstar LyoQuest Plus freeze dryer, equipped with a vacuum pump system (Telstar 2G‐6, Spain). The condenser temperature was set at −40.8°C, with a vacuum level of 0.62 mbar, for a duration of 72 h. Following the freeze‐drying process, the samples were collected, finely powdered using a high‐speed grinder, and were immediately transferred into airtight containers and stored within a desiccator containing blue silica gel at 25^°^C ± 1^°^C under dark conditions until further analysis. This storage environment maintains a relative humidity (RH) below 20% to prevent moisture uptake.

The freeze‐drying yield was determined using Equation (1), which calculates the product recovery as the ratio of the mass of the recovered product to the mass of the product fed into the system (on a dry basis):

(1) Product yield %=the weight of obtained dried powderthe total solid weight in initial feed ×100

### 2.4. Determination of Composite Particulate System Properties

#### 2.4.1. Moisture Content (MC) and Water Activity

The MC of the dry particulates was determined using the gravimetric hot air oven drying method. A known sample weight of 0.5 g was dried at 105°C until a constant weight was achieved. The MC was calculated based on the initial and final powder weights using Equation (2) and was expressed on a wet basis:

(2) MC %wb=W1−W2W1×100

where *W*_1_ is the weight before drying (gram) and *W*_2_ is the weight after drying (gram).

The water activity (*a*_w_) of the dry particulates was determined using an electronic dew point water activity meter (Aqualab Dew Point 4TE).

#### 2.4.2. Particulate Density and Flowability

The bulk density was calculated by weighing 2 g of the dry particulates into a graduated 10‐mL cylinder and measuring the volume occupied by the sample. For tapped density, the cylinder was tapped manually for 5 min at a rate of 32 taps/min, and the volume occupied by the sample was measured in the same manner as for bulk density.

The Hausner ratio (HR) and the Carr index (CI) are important properties used to assess the flowability of the dry particulates [21]. HR indicates the cohesiveness of the dry particulates and was calculated as the ratio of tapped density to bulk density Equation (3), while CI represents the powders’ flowability and was determined using Equation (4):

(3) HR=ρtρb

(4) CI %=100×1−1HR

where *ρ*_*t*_ is the tapped density of dry particulates (g/cm^3^) and *ρ*_*b*_ is the bulk density of dry particulates (g/cm^3^).

Values of cohesiveness allow classifying the particles in terms of HR: > 1.4 = high, 1.2–1.4 = intermediate, and < 1.2 = low. The flowability of the powders was classified in terms of CI (%): < 15 = very good, 15–20 = good, 20–35 = fair, 35–45 = bad, and > 45 = very bad [22].

#### 2.4.3. Water Solubility, Wettability, Dispersibility, and Hygroscopicity

##### 2.4.3.1. Water Solubility

The water solubility of the dry particulates was determined following the method of Santhalakshmy et al. [22]. Briefly, 1 g (on a dry basis) of the particulates was added to 100 mL of distilled water in a high‐velocity blender and mixed for 5 min. After mixing, the suspension was centrifuged at 3000 rpm for 5 min, yielding a supernatant. A 25‐mL aliquot of the supernatant was then dried using a hot air oven (Macro Scientific Works Pvt. Ltd., RM‐SP‐325, India) at 105°C for 5 h in preweighed Petri dishes. The solubility percentage was calculated based on the weight difference before and after drying.

##### 2.4.3.2. Wettability

Wettability was measured using the method of Vissotto et al. [23]. A glass funnel was positioned 10 cm above the water surface, supported by a ring stand. Inside the funnel, a test tube containing 1 g of powder was surrounded by the sample. The test tube was then lifted gradually, starting a stopwatch simultaneously. The wettability time was recorded when all powder particles had visibly passed through the water’s surface.

##### 2.4.3.3. Dispersibility

For measurement of dispersibility, the method described by Santhalakshmy et al. [22] was used. Briefly, a 50‐mL beaker containing 10 mL of distilled water at 25°C was used, to which 1 g of powder was added and vigorously agitated for 15 s, completing 25 full back‐and‐forth movements across the beaker’s diameter. After agitation, the mixture was sieved through a 212‐*μ*m sieve. The powder that remained on the sieve was then transferred onto a preweighed, predried aluminum pan and dried for 4 h in a hot air oven at 105°C. Dispersibility was calculated using Equation (5):

(5) Dispersibility %=10+a×%TSa×100−b/100

where *a* is the quantity of powder used (gram), *b* is the amount of moisture in the powder, and *%*TS is the percentage of dry matter in the reconstituted juice after it has been sieved.

##### 2.4.3.4. Hygroscopicity

The hygroscopicity of the particulate system was evaluated by measuring its moisture uptake, following a modified methodology [24]. Specifically, 1 g of sample was placed in a glass desiccator maintained at 25°C. The desiccator contained a saturated NaCl solution, ensuring a RH of 75.29%. After a 1‐week incubation period, the samples were carefully weighed, and hygroscopicity was calculated using Equation (6):

(6) Hygroscopicity=final weight of the dried sample−initial weight of the sampleinitial weight ×100

#### 2.4.4. Proximate Analysis

The compositional profile of vegetable–fruit–herb particulate system samples, specifically their protein, fat, and ash content, was assessed using AOAC methodologies [25]. After quantifying fat, protein, and ash content, the total carbohydrate was calculated using Equation (7):

(7) Carbohydrate %=100−protein+fat+ash+water%

#### 2.4.5. Color Analysis

A handheld chromameter (CR‐400, Konica Minolta, Japan) operating on the Hunter color scale was employed to measure the color of the freeze‐dried powder. The instrument was first calibrated using standard white tiles to ensure accuracy. Following calibration, the color parameters lightness (*L* ^∗^), redness (*a* ^∗^), and yellowness (*b* ^∗^) were recorded.

#### 2.4.6. Morphological Characterization

The morphological characteristics of the particulate system were analyzed using a scanning electron microscope (JEOL, JSM‐6510 SEM) as described by Ghanghas et al. [26]. A small amount of the well‐mixed sample was carefully extracted and coated with a thin layer of gold under high‐vacuum conditions to enhance electron beam reflection. SEM images were captured at an accelerating voltage of 15 kV with a magnification of 100× and 250×. The shape, size, and surface properties of the particles in each sample were examined and documented.

#### 2.4.7. Functional Group Analysis

An attenuated total reflection Fourier‐transform infrared (ATR‐FTIR) spectrophotometer (Tensor 27, Bruker Corp, Massachusetts, United States) was utilized to analyze the functional groups of the particulate system at room temperature. Before each analysis, the ATR plate was thoroughly cleaned with analytical‐grade isopropyl alcohol and dried to ensure accuracy. A background scan was performed prior to sample analysis. The samples were then directly placed on the ATR plate, and FTIR analysis was conducted. Sixteen scans at a resolution of 2 cm^−1^ were performed, covering the IR range of 4000–400 cm^−1^ to characterize the primary functional groups. The spectral data were analyzed using BRUKER Software Version 7.5 (1991) (OriginLab Corporation, Northampton, Massachusetts, United States).

#### 2.4.8. Thermal Properties

The glass transition temperature of the particulate system was determined using a differential scanning calorimeter (DSC 200, NETZSCH, Germany), following the methodology described by Sharanagat et al. [27]. Approximately 0.5 mg of each sample (dry weight basis) was accurately weighed into aluminum crucibles with a capacity of 40 *μ*L and hermetically sealed. An empty aluminum crucible served as the reference. The thermal analysis protocol began with a 5‐min equilibration period at 25°C, followed by heating from 25°C to 200°C at a constant rate of 10°C per minute under a nitrogen purge of 20 mL/min to prevent oxidative degradation. Heat flow data were recorded at 0.5‐s intervals throughout the heating phase. The glass transition was identified as an endothermic shift in the baseline of the heat flow curve. Using NETZSCH Proteus Analysis software, three characteristic temperatures were determined: the onset temperature (*T*_o_), defined as the intersection point of the extrapolated pretransition baseline and the tangent at the inflection point; the peak temperature (*T*_p_), corresponding to the maximum heat flow deviation within the transition region; and the end temperature (*T*_e_), identified where the curve converged with the extrapolated posttransition baseline. Triplicate measurements were performed for each sample, and average values are reported.

Additionally, thermal conductivity (W/mK), thermal diffusivity (mm^2^/s), and volumetric heat capacity (MJ/m^3^K) were measured using the Hot Disk TPS 500 S instrument (Hot Disk AB, Göteborg, Sweden), following the methodology described by [21].

#### 2.4.9. Antioxidant Properties

##### 2.4.9.1. Total Phenolic Content (TPC) and Total Flavonoid Content (TFC)

The TPC of the particulate system was determined using the FC reagent, as described by Singleton and Rossi [28] with minor modifications. Briefly, diluted samples (1:1) were mixed with 0.5 mL of FC reagent. After 3 min, 0.5 mL of 7.5% sodium carbonate solution was added, and the mixture was diluted to a total volume of 5 mL with distilled water. The solution was incubated in the dark at 25°C for 2 h. Following incubation, the absorbance was measured at 765 nm using a UV–vis spectrophotometer (MutSpec–1501, Shimadzu, Japan). Gallic acid (0–100 mg/mL) was used as a standard. The TPC results were expressed as milligrams of gallic acid equivalent per g (mg GAE/g) of sample, based on the average of three independent replicates.

The TFC was determined following the described method of Sharanagat et al. [27] with slight adjustments. A 1‐mL sample was mixed with 3 mL of distilled water, followed by the addition of 0.30 mL of 5% NaNO_2_ solution. After 5 min of incubation at 25°C, 0.30 mL of 10% AlCl_3_ solution was added. Following another 5 min, 2 mL of 1 M NaOH solution was introduced. The reaction mixture was diluted with 10 mL of distilled water, and its absorbance was measured at 510 nm using a spectrophotometer (MutSpec–1501, Shimadzu, Japan). Quercetin served as the standard, and TFC values were reported as milligrams of quercetin equivalents (QE) per gram of sample.

##### 2.4.9.2. DPPH Assay

The antioxidant activity of the samples was evaluated using the DPPH radical scavenging assay, as previously described by Suhag et al. [29] with slight modifications. A 0.1 mM DPPH solution was prepared by dissolving DPPH in methanol to create a stock solution. For the assay, 0.1 mL of the sample extract was mixed with 3.9 mL of the DPPH stock solution. The mixture was incubated in the dark at 25°C for 1 h. A control solution was prepared by replacing the sample extract with 0.1 mL of methanol. After the incubation period, the absorbance was measured at 517 nm using a UV–vis spectrophotometer (MutSpec–1501, Shimadzu, Japan). The DPPH radical scavenging activity was determined using Equation (8):

(8) Scavenging activity %=1−A1A0×100

where *A*_0_ is the absorbance of a control solution and *A*_1_ is the absorbance of samples.

#### 2.4.10. Sensory Analysis

A precise quantity (11.5 g) of the sample was weighed and transferred to a cup. To achieve a total weight of 100 g, 88.5 g of water was added. The mixture was gently stirred or shaken to ensure complete dissolution of the powder and uniform distribution of its particles throughout the solution. The reconstituted beverage was deemed ready for evaluation once the powder was fully dissolved and the mixture exhibited a visually uniform appearance.

Consumer acceptability was assessed using the hedonic scale rating method. A questionnaire was developed to evaluate panelists’ (*n* = 30; male = 14 and female = 16; age range = 20–40 years) acceptance and perceptions of the instant powdered beverage mix. The questionnaire was divided into five sections. Panelists rated their satisfaction with attributes such as appearance, texture, aroma, and overall acceptability in the first four sections. The fifth section included a single question regarding aftertaste, where panelists provided a “yes” or “no” response.

### 2.5. Statistical Analysis

Each measurement for every sample was conducted in triplicate. The results are presented as means and standard deviations. The significance of differences between the mean values of the properties of the freeze‐dried juice powders was assessed using one‐way analysis of variance (ANOVA) followed by Tukey’s test (*p* ≤ 0.05). Data analysis was performed using Minitab Software (Version 21.1.0).

## 3. Result and Discussion

### 3.1. Product Yield, MC, and Water Activity

The selected freeze‐drying process conditions resulted in a high product yield (Figure 2a). The results showed that all four samples achieved good yields, ranging from 92.92% to 94.55%, with Sample S1 exhibiting the highest yield. These findings highlight the effectiveness of the freeze‐drying process in preserving fruit juice while retaining a substantial portion of the original solids content. Similar yields of over 90% have been reported for strawberry, mango, and orange juice powders produced using spray‐drying [30] and yields of around 80% for blends of red grape, mulberry, and strawberry obtained via freeze‐drying [31]. However, variations in the yield of different particulate systems can be attributed to differences in carrier agent composition, juice type, and process conditions.

Figure 2(a) Yield, (b) moisture content, (c) water activity, (d) bulk and tapped density, (e) Hausner ratio, and (f) Carr index of the particulate system. For Sample IDs S1, S2, S3, and S4, please refer to Table 1. Different superscript letters indicate significant differences (*p* < 0.05). For density, lowercase letters refer to bulk density and uppercase letters refer to tapped density.

**Figure:**
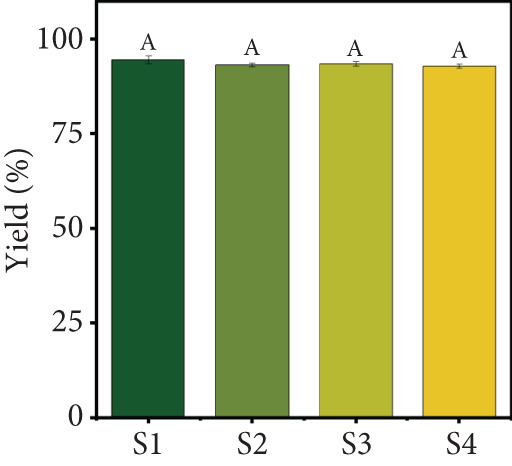
(a)

**Figure:**
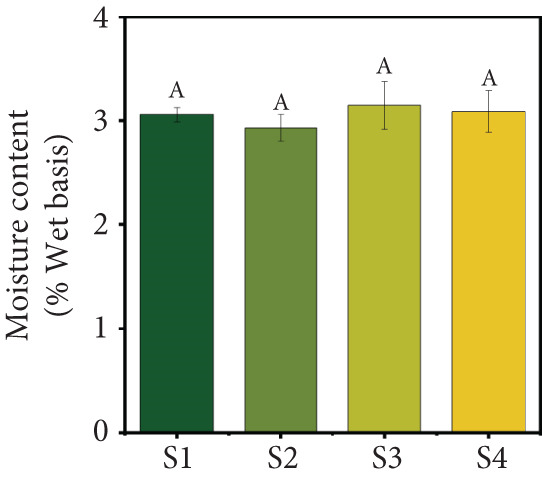
(b)

**Figure:**
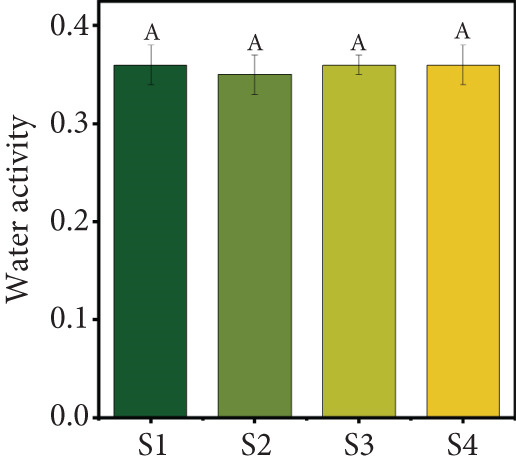
(c)

**Figure:**
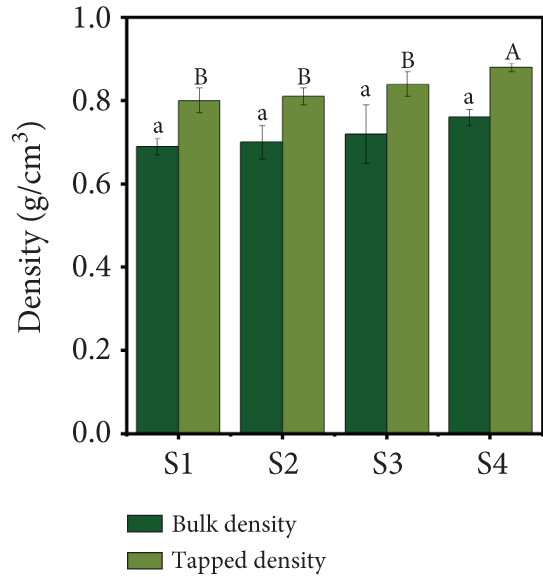
(d)

**Figure:**
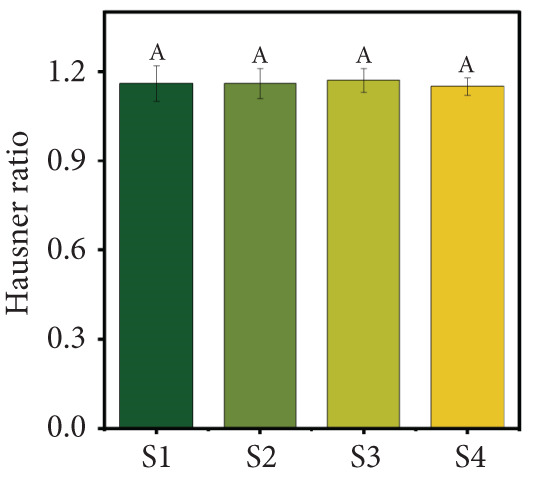
(e)

**Figure:**
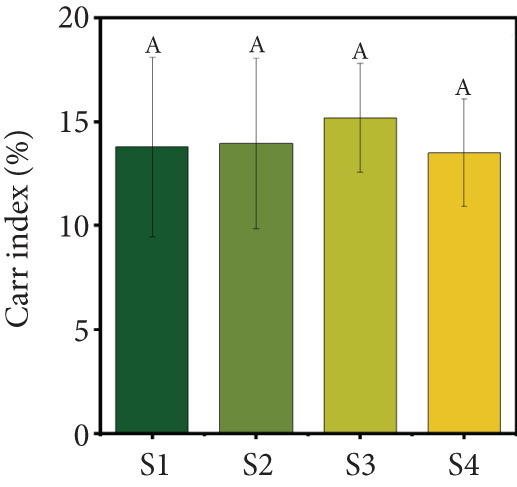
(f)

MC and water activity (*a*_w_) are critical factors affecting the stability and quality of particulate systems. In this study, the MC and *a*_w_ values were not significantly different (*p* > 0.05) across the four particulate systems (Figure 2b,c). The MC ranged from 2.93% to 3.15% (w.b.), while the *a*_w_ ranged from 0.35 to 0.36. Low water activity is particularly important for inhibiting the growth of most bacteria, yeasts, and molds, which are unable to thrive below water activity levels of 0.87, 0.88, and 0.80, respectively. The MC and *a*_w_ values observed in this study fall within the ranges reported in previous research [30, 32, 33].

### 3.2. Density and Flowability

The results of the bulk and tapped density measurements for different particulate systems are presented in Figure 2d. Sample S4 exhibited the highest bulk density (0.76 ± 0.02 g/cm^3^), followed by Samples S3 (0.72 ± 0.07 g/cm^3^), S2 (0.70 ± 0.04 g/cm^3^), and S1 (0.69 ± 0.02 g/cm^3^). Similarly, the tapped density followed the same trend: Sample S4 had the highest value (0.88 ± 0.01 g/cm^3^), followed by S3, S2, and S1. The average tapped density across all four samples was 0.83 g/cm^3^. Various factors influence a powder tapped density, including particle size, shape, surface roughness, MC, and particle size distribution [17]. The observed density values were similar to those reported for guava powder [12] but higher than those reported for beetroot [34, 35], blueberry, carrot, cranberry [35], and Andean blueberry powders [36]. These differences may be attributed to the use of a blend of guava, beetroot, orange, and mint juices, which likely formed a complex particulate system with denser particles.

The HR and CI are widely recognized indicators of powder flowability [17]. In this study, the HR (Figure 2e) and CI (Figure 2f) values did not show significant differences (*p* > 0.05) across the four powder samples. This suggests that the different formulations of the particulate systems did not significantly affect flowability. The HR values ranged from 1.15 to 1.17, while the CI values ranged from 13.51% to 15.18%. These results indicate that the freeze‐dried particulate complex exhibits very good flowability and low cohesiveness, based on CI and HR values, respectively. Comparable CI (13%–27%) and HR (1.169–1.377) values have been reported for beetroot, blueberry, carrot, and cranberry powders [35]. In contrast, guava powder has been reported to have higher CI (23.5%) and HR (1.31) values [12]. The lower values observed in this study may be attributed to the blend of different juices, which likely resulted in a more uniform particulate system with improved flow properties.

### 3.3. Solubility, Wettability, Dispersibility, and Hygroscopicity

The solubility of a powder is a critical parameter that characterizes its wettability and dispersibility in solutions [37]. It also determines how easily the powder can be reconstituted in water at room temperature [38]. The water solubility, wettability, and dispersibility of freeze‐dried instant beverage mix powder samples are presented in Table 2. Water solubility did not show significant differences (*p* > 0.05) across the four samples, with values ranging from 88.08% to 89.72%. In comparison, freeze‐dried Andean blueberry juice powders demonstrated higher water solubility (around 93%), unaffected by variations in maltodextrin concentration (30%–50%) [36]. Similarly, nearly 100% solubility was reported for blackberry juice powders produced via freeze‐ and spray‐drying [39]. Factors influencing the solubility of freeze‐dried powders include particle morphology, size, interparticle voids, and the properties of the juice and carrier agents used [17].

**Table 2:** Solubility, hygroscopicity, wettability, dispersibility, and proximate composition of particulate systems.

**Sample**	**Solubility (%)**	**Wettability (s)**	**Dispersibility (%)**	**Hygroscopicity (%)**	**Fat (%)**	**Protein (%)**	**Ash (%)**	**Carbohydrates (%)**
S1	89.72 ± 6.68^a^	112.33 ± 2.52^b^	93.50 ± 0.73^a^	30.65 ± 0.02^d^	0.83 ± 0.04^a^	2.91 ± 0.19^a^	2.50 ± 0.22^a^	90.36 ± 0.28^a^
S2	88.74 ± 6.36^a^	116.66 ± 1.52^ab^	91.93 ± 1.63^ab^	32.18 ± 0.05^c^	0.87 ± 0.02^a^	2.61 ± 0.17^a^	2.56 ± 0.12^a^	90.75 ± 0.30^a^
S3	88.08 ± 11.31^a^	121.33 ± 3.51^a^	89.78 ± 1.06^bc^	32.61 ± 0.05^b^	0.88 ± 0.02^a^	2.38 ± 0.27^a^	2.63 ± 0.09^a^	90.69 ± 0.33^a^
S4	88.44 ± 1.41^a^	123.67 ± 4.04^a^	87.19 ± 1.82^c^	34.46 ± 0.04^a^	0.88 ± 0.03^a^	2.43 ± 0.29^a^	2.76 ± 0.10^a^	90.72 ± 0.25^a^

*Note:* Different superscript letters in the same column indicate significant differences (*p* < 0.05).

Wettability values ranged from 112.33 ± 2.52 to 123.67 ± 4.04 s, with Sample S4 exhibiting the highest wettability time, followed by S3, S2, and S1 (Table 2). The higher wettability times may be attributed to increased sugar content, which enhances water molecule attraction and binding. The hygroscopic nature of sugars facilitates water absorption, leading to longer wetting times [40].

Dispersibility values ranged from 87.19*%* ± 1.82*%* to 93.50*%* ± 0.73*%*, with Sample S1 showing the highest dispersibility, followed by S2, S3, and S4 (Table 2). Coarse and uneven particle surfaces enhance water penetration, improving dispersibility. Morphological images revealed highly uneven particles with angular structures, and circularity analysis indicated that as the particle circularity and convexity increase, the percentage of undissolved particles also rises. Conversely, larger particle areas generally correspond to lower percentages of undissolved particles [41].

Hygroscopicity, which refers to a product’s ability to absorb moisture from the environment, is an essential quality indicator for powdered products. Low hygroscopicity suggests better powder stability and flowability during storage [42]. In this study, hygroscopicity values ranged from 30.65*%* ± 0.02*%* to 34.46*%* ± 0.04*%* (Table 2), with statistically significant differences among the samples (*p* < 0.05). Sample S1 exhibited the lowest hygroscopicity, followed by S2, S3, and S4, indicating that S1 is the most stable under high humidity conditions. For comparison, Kapoor and Feng [35] reported hygroscopicity values of 33.55% and 31.50% for freeze‐dried cranberry and blueberry powders, respectively.

### 3.4. Proximate Composition

The fat, protein, ash, and carbohydrate contents of the particulate systems were calculated, and the results are summarized in Table 2. The fat, protein, ash, and carbohydrate levels across the four samples ranged from 0.83% to 0.88%, 2.38%–2.91%, 2.50%–2.75%, and 90.36%–90.75%, respectively, with no statistically significant differences (*p* > 0.05) observed among them. The proximate composition reported in our study is in line with the values reported by Saifullah et al. [12] for commercial pitaya, pineapple, mango, and guava fruit powders.

### 3.5. Color Profile

The CIELAB color coordinates of the freeze‐dried juice powder samples are presented in Table 3. The lightness (*L* ^∗^) values increased significantly (*p* < 0.05) from 26.93 ± 0.19 to 31.78 ± 0.58 across Samples S1 to S4. Conversely, the redness (*a* ^∗^) values decreased significantly (*p* < 0.05) from 31.77 ± 0.68 to 27.04 ± 0.43. This increase in lightness and decrease in redness can be attributed to the progressive reduction in the concentration of beetroot used in preparing samples from S1 to S4. The *b* ^∗^ values did not differ significantly (*p* > 0.05) among the samples. The hue angle (*h*) and chroma are key attributes defining color perception and intensity, respectively. Chroma values decreased significantly (*p* < 0.05) from 32.17 ± 0.68 to 27.47 ± 0.41 across Samples S1 to S4. The color profile values observed in this study are within the range reported in previous studies on various fruit juice powders [6, 30]. Consistent with the nature of these products, all powders exhibited low hue angle (*h*) values, indicating proximity to the red spectrum (0°). Similarly, Estupiñan‐Amaya et al. [36] reported low *h* values (2.2°–5.5°) for freeze‐dried Andean blueberry juice powders.

**Table 3:** Color profile and morphological characterization of composite particulates.

**Sample**	**Color profile**					**Morphological characterization**		
	**L** ^∗^	**a** ^∗^	**b** ^∗^	**Chroma**	**h** ^∗^	**Area (*μ*m** ^**2**^ **)**	**Perimeter (*μ*m)**	**Circularity**
S1	26.93 ± 0.19^d^	31.77 ± 0.68^a^	5.01 ± 0.17^a^	32.17 ± 0.68^a^	0.15 ± 0.01^a^	115,210	1430.79	0.71
S2	27.86 ± 0.28^c^	30.32 ± 0.39^b^	4.85 ± 0.94^a^	30.71 ± 0.40^b^	0.15 ± 0.02^a^	77,563.5	1259.83	0.61
S3	29.26 ± 0.45^b^	27.97 ± 0.19^c^	4.98 ± 0.13^a^	28.41 ± 0.23^c^	0.17 ± 0.01^a^	79,181.4	1358.57	0.54
S4	31.78 ± 0.58^a^	27.04 ± 0.43^d^	4.90 ± 0.11^a^	27.47 ± 0.41^d^	0.17 ± 0.01^a^	58,536.6	1166.18	0.54

*Note:* Different superscript letters in the same column indicate significant differences (*p* < 0.05).

### 3.6. Morphological Characterization

**Figure 3:**
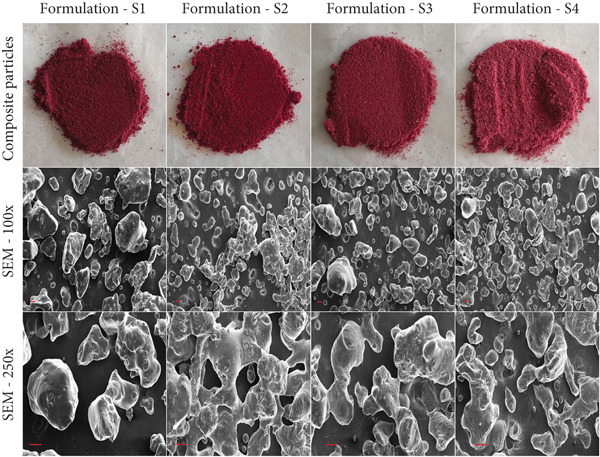
Produced composite particles and their scanning electron micrograph at 100× and 250×. Scale refers to 100 *μ*m.

The morphological characteristics of the composite particulates were analyzed using scanning electron microscopy (SEM). Key parameters, including area, perimeter, and circularity, for the instant beverage mix powder samples are detailed in Table 3. SEM micrographs at 100× and 250× magnifications for the four samples are shown in Figure 3. At 100× magnification, Sample S1 displayed irregularly shaped particles of varying sizes, which transitioned to smoother surfaces at 250× magnification. Sample S2 exhibited more irregular and coarser particles, with a porous structure that became more pronounced at higher magnification. Sample S3 revealed a heterogeneous mix of smooth and rough particles, along with partially fused spherical shapes, indicating compositional diversity. Sample S4 showed a highly rough and irregular morphology with significant porosity, suggesting a complex surface topology. These observations align with findings from [37], who reported that freeze‐dried pomegranate juice powders prepared with maltodextrin and gum Arabic predominantly exhibited angular shapes, while those prepared with waxy starch displayed more uniform, spherical morphologies.

Furthermore, morphological analysis, along with other physical and functional analyses, offered critical insights into the functional behavior of the particulate systems. For example, in Figure 3 at 100× magnification, it can be observed that Sample S1 exhibited a greater number of free, irregular particles with larger surface areas (115,210 *μ*m^2^, Table 3) and reduced aggregation, directly contributing to its superior solubility (89.72%) and dispersibility (93.50%) (Table 2). The increased surface roughness and particle separation facilitated rapid water penetration during reconstitution. Concurrently, S1’s lower hygroscopicity (30.65%) correlated with its morphological structure: larger particles with smoother surfaces minimized moisture adsorption sites compared to S4’s smaller, porous particles (58,536.6 *μ*m^2^ and hygroscopicity: 34.46%), supported by the strong negative correlation between particle area and hygroscopicity (*r* = −0.66, correlation data not shown). However, flowability remained consistent across samples (CI: 13.51%–15.18%; HR: 1.15–1.17) despite morphological variations, as uniform particle density (0.69–0.76 g/cm^3^) and low MC (< 3.2%) dominated flow behavior by minimizing cohesive forces. Hygroscopicity differences were further amplified by compositional factors, evidenced by its strong positive correlation with sugar‐rich components (e.g., orange content: *r* = 0.92). These observations emphasize that powder flowability is holistically governed by multiple factors, including MC, particle geometry, and cohesiveness, necessitating integrated optimization [17]. Thus, while morphology primarily modulates dissolution and moisture affinity, flow stability emerges from synergistic physical factors, with compositional gradients exerting a dominant influence on hygroscopic responses in complex blends.

### 3.7. Functional Group Analysis

**Figure 4:**
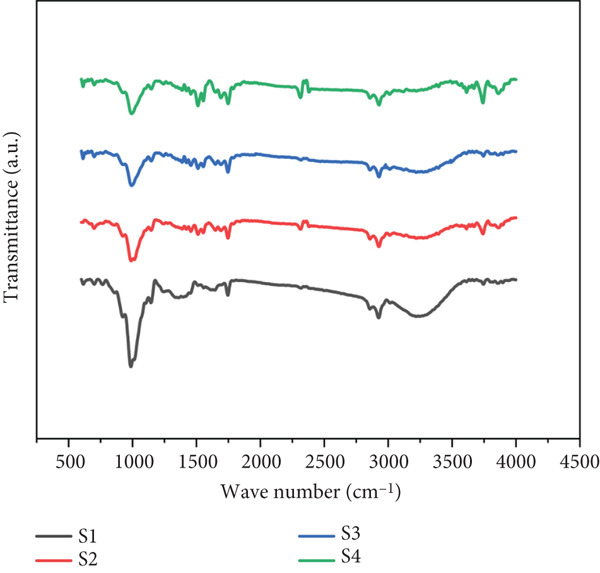
FTIR spectrum of the composite particles. For Sample IDs S1, S2, S3, and S4, refer to Table 1.

The FTIR spectra of the composite particulate systems (S1–S4) revealed characteristic absorption bands corresponding to major biomolecular constituents (Figure 4). The broad peak at 3280–3350 cm^−1^ (O‐H/N‐H stretching) indicated hydrogen‐bonded hydroxyl groups from polysaccharides, polyphenols, and residual moisture. Distinct protein signatures were observed at 1689.88 cm^−1^ (amide I: C=O stretching) and 1530–1550 cm^−1^ (amide II: N‐H bending/C‐N stretching), consistent with fruit juice powder profiles reported in the literature [43]. Carbohydrate regions showed prominent peaks at 1014–1100 cm^−1^ (C‐O‐C/C‐O glycosidic bonds), 989.24 cm^−1^ (C‐O stretching in polysaccharides), and 924.99 cm^−1^ (pyranose ring vibrations), confirming maltodextrin and fruit‐derived carbohydrate retention. Sugar‐specific bands appeared at 1150 cm^−1^ (C‐O stretching in sucrose/fructose) and 1050 cm^−1^ (C‐O‐H bending). Crucially, polyphenol fingerprints were identified at 1750 cm^−1^ (esterified C=O in phenolic acids), 1600–1630 cm^−1^ (aromatic C=C stretching in flavonoids), and 1270 cm^−1^ (C‐O stretching in phenolic compounds), aligning with reference spectra for guava phenolics and beetroot betalains. While the spectral homogeneity across samples suggests compositional stability, FTIR provides functional group‐level insights rather than compound‐specific quantification. Complementary quantitative analyses (Table 2: carbohydrates 90.36%–90.75%; phenolics 101–160 mg GAE/100 g) support these molecular assignments and confirm bioactive retention.

### 3.8. Thermal Properties

**Figure 5:**
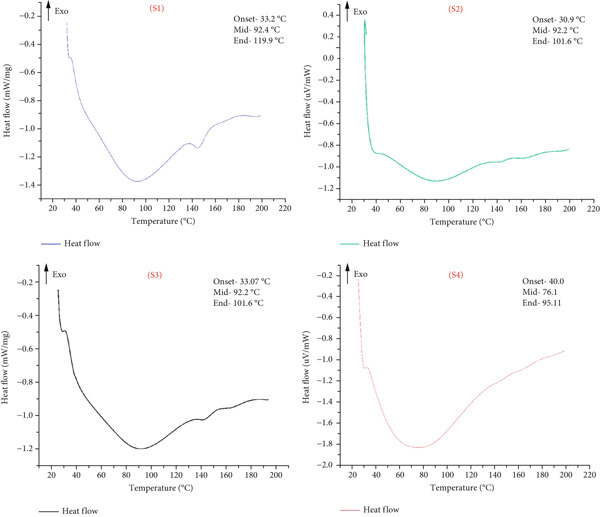
DSC thermograms of the composite particles. For Sample IDs S1, S2, S3, and S4, refer to Table 1.

The thermal properties of the complex particulates, including thermal conductivity (*λ*), thermal diffusivity (*α*), and volumetric specific heat (*ρ*_cp_), were evaluated and are summarized in Table 4. These properties are essential for understanding heat transfer and energy requirements in food processing and packaging [21]. The thermal conductivity values ranged from 0.13 to 0.14 W/mK, indicating no significant difference (*p* > 0.05) across the samples. Thermal diffusivity and volumetric specific heat exhibited minor but statistically significant variations (*p* < 0.05). Thermal diffusivity ranged from 0.12 ± 0.02 to 0.16 ± 0.01 mm^2^/s, while volumetric specific heat ranged from 0.80 ± 0.01 to 1.18 ± 0.02 MJ/m^3^K. Notably, Sample S4 exhibited the highest thermal diffusivity, while Sample S1 showed the highest volumetric specific heat. These differences are likely due to variations in the composition, particle size, and MC of the powders. The specific contributions of sugars, acids, and other ingredients can significantly influence the thermal properties of the fruit juice powders, with higher thermal diffusivity indicating a more efficient heat transfer, which is beneficial for processes requiring rapid and uniform heating or cooling. The glass transition temperature (*T*_g_), a key parameter that marks the transition from a rigid, glassy state to a more flexible, rubbery state, was investigated using differential scanning calorimetry (DSC). The results, shown in Table 4 and Figure 5, revealed distinct thermal behaviors among the samples. Sample S1 exhibited a broad glass transition range with an onset temperature of 33.2^°^C ± 0.1^°^C, a midpoint of 92.2^°^C ± 0.2^°^C, and an end temperature of 119.9^°^C ± 0.1^°^C, indicating a gradual transition from a glassy to a rubbery state. Sample S2, on the other hand, had a slightly lower onset temperature of 30.9^°^C ± 0.1^°^C, the same midpoint at 92.2^°^C ± 0.1^°^C, but completed the transition sooner with an end temperature of 101.6^°^C ± 0.1^°^C, suggesting higher mobility of the polymer chains or compositional differences that affect thermal behavior. Sample S3 displayed thermal characteristics not significantly different from S2, with an onset of 33.1^°^C ± 0.1^°^C, a midpoint of 92.2^°^C ± 0.1^°^C, and an end at 101.6^°^C ± 0.1^°^C, indicating a consistent thermal profile and material composition. In contrast, Sample S4 exhibited a distinct profile, with a higher onset temperature of 40.0^°^C ± 0.2^°^C, a lower midpoint of 76.1^°^C ± 0.1^°^C, and an earlier end temperature of 95.1^°^C ± 0.1^°^C, suggesting a delayed onset but a quicker completion of the glass transition. This behavior likely reflects differences in molecular structure or composition that influence chain mobility and flexibility. The variations in the glass transition temperature and other thermal parameters among the samples highlight the impact of molecular structure and material composition on the thermal stability of the powders. Understanding these thermal properties is crucial for optimizing processing and storage conditions, ensuring the stability and functional integrity of freeze‐dried fruit juice powders in various applications.

**Table 4:** Thermal properties of composite particulates.

**Sample**	**Thermal properties**					
	**T**_**o**_ **(°C)**	**T**_**p**_ **(°C)**	**T**_**e**_ **(°C)**	**Thermal conductivity (W/mK)**	**Thermal diffusivity (mm** ^**2**^ **/s)**	**Volumetric heat capacity (MJ/m** ^**3**^ **K)**
S1	33.2 ± 0.1^b^	92.4 ± 0.2^a^	119.9 ± 0.1^a^	0.14 ± 0.01^a^	0.12 ± 0.02^c^	1.18 ± 0.02^a^
S2	30.9 ± 0.1^c^	92.2 ± 0.1^a^	101.6 ± 0.1^b^	0.13 ± 0.02^a^	0.14 ± 0.01^b^	0.94 ± 0.01^c^
S3	33.1 ± 0.1^b^	92.2 ± 0.1^a^	101.6 ± 0.1^b^	0.14 ± 0.01^a^	0.13 ± 0.01^c^	1.05 ± 0.03^b^
S4	40.0 ± 0.2^a^	76.1 ± 0.1^b^	95.1 ± 0.1^c^	0.13 ± 0.01^a^	0.16 ± 0.01^a^	0.80 ± 0.01^d^

*Note:* Different superscript letters in the same column indicate significant differences (*p* < 0.05).

Abbreviations: *T*_e_, end temperature; *T*_o_, onset temperature; *T*_p_, peak temperature.

### 3.9. Antioxidant Properties

The TPC, TFC, and DPPH radical scavenging ability of the freeze‐dried fruit, vegetable, and herb juice powder samples were evaluated. The results are presented in Table 5. TPC values ranged from 101.81 ± 7.20 to 160.11 ± 6.30 mg GAE/100 g, with Sample S4 exhibiting the highest value, followed by S3, S2, and S1. These differences highlight the influence of fruit composition and proportions on phenolic content. Specifically, the ratios of guava, beetroot, orange, and mint in the formulations played a significant role. For comparison, freeze‐dried guava extracts typically exhibit TPC values of 295.30 mg GAE/100 g [44], beetroot powder ranges from 80 to 320 mg GAE/100 g [6], and orange powder has a TPC of 346 mg GAE/100 g [45]. These variations underscore the diverse phenolic profiles contributed by individual fruit powders.

**Table 5:** Total phenolic content (TPC), DPPH radical scavenging activity, and total flavonoid content (TFC) of composite particulates.

**Sample**	**Antioxidant properties**		
	**TPC (mg GAE/100** g**)**	**DPPH (%)**	**TFC (mg QE/g)**
S1	101.81 ± 7.20^d^	59.33 ± 1.36^a^	158.02 ± 0.97^a^
S2	117.73 ± 6.76^c^	56.81 ± 0.70^b^	140.58 ± 2.01^b^
S3	141.84 ± 4.92^b^	49.48 ± 0.18^c^	133.87 ± 1.21^c^
S4	160.11 ± 6.30^a^	46.91 ± 0.25^d^	126.01 ± 0.36^d^

*Note:* Different superscript letters in the same column indicate significant differences (*p* < 0.05).

The TFC values ranged from 126.01 ± 0.36 to 158.02 ± 0.97 mg QE/g, with Sample S1 showing the highest content, followed by S2, S3, and S4. The differences in TFC are attributed to the intrinsic flavonoid levels of the individual fruits and the proportions used in the formulations. Previous studies report TFC values of 96.93–105.07 mg QE/g for guava powder [44], 7.04 mg QE/g for beetroot powder [46], and 34.16 mg QE/100 g for orange juice powder [47]. The decrease in flavonoid content across Samples S1 to S4 is likely due to the reduced beetroot content, increased orange proportion, and higher mint content in the formulations. Mint generally has a lower flavonoid concentration compared to the other fruits in this study.

The DPPH radical scavenging ability, a measure of the antioxidant activity, ranged from 46.91*%* ± 0.25*%* to 59.33*%* ± 1.36*%*, with Sample S1 exhibiting the highest activity and Sample S4 the lowest. These variations align with the antioxidant capacities of the individual fruit powders and their proportions in the formulations. Guava powder has a reported DPPH activity of 88.1% [48], 95.31% for beetroot powder [49], and ~60% for citrus powder [50]. The decline in DPPH activity from S1 to S4 corresponds to the decreasing proportion of beetroot powder and the increasing ratio of orange powder in the formulations. The lower antioxidant capacity of orange powder relative to beetroot powder likely contributed to this trend.

These findings demonstrate that the phenolic and flavonoid contents, as well as antioxidant activities, are heavily influenced by the compositional ratios of fruit powders in the freeze‐dried samples. The adjustment of these proportions can modulate the bioactive properties, offering potential for tailoring the nutritional and functional attributes of instant beverage powders.

### 3.10. Consumer Acceptability

**Figure 6:**
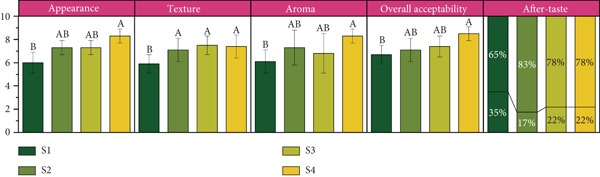
Sensory analysis of the composite particulate system. For after‐taste, the upper % refers to “no” and lower to “yes.” Different superscript letters indicate significant differences (*p* < 0.05).

The sensory evaluation of the four composite particulate samples, focusing on appearance, texture, aroma, and overall acceptability, revealed significant variations (Figure 6). Among the samples, S4 achieved the highest scores across all categories, with an appearance rating of 8.3, a texture score of 7.4, an aroma rating of 8.3, and an overall acceptability of 8.5. These ratings indicate that S4 possessed a superior sensory profile, making it the most preferred among the tested samples. The balanced combination of ingredients in S4 likely contributed to its enhanced sensory appeal, particularly in appearance and aroma. Samples S2 and S3 displayed similar sensory attributes, with S3 slightly outperforming S2 in texture (7.5 vs. 7.1) and overall acceptability (7.4 vs. 7.1). Both samples shared an appearance rating of 7.3, indicating visual appeal. However, S3’s slightly lower aroma score of 6.8 did not substantially impact its overall acceptability. These findings suggest that while both samples were well received, slight differences in ingredient composition or processing might have influenced their textural and aromatic characteristics. In contrast, Sample S1 received the lowest scores in all sensory categories, particularly for texture (5.9) and appearance (6.0), resulting in an overall acceptability score of 6.7. These results suggest that formulation may have contributed to its less favorable sensory attributes, making it the least preferred among the samples.

Overall, these findings highlight the importance of optimizing sensory attributes, such as texture, aroma, and visual appeal, to enhance consumer acceptability. The consistent high ratings of S4 across all parameters highlight its potential for commercial applications, while the lower scores of S1 emphasize the need for formulation adjustments to improve its sensory characteristics.

## 4. Conclusion

This study successfully developed freeze‐dried polyphenol‐rich composite particulates from guava, beetroot, orange, and mint without added preservatives, sugars, or artificial sweeteners. The particulate system demonstrated low MC and water activity, suggesting potential for long shelf life and microbiological stability. Physicochemical analyses revealed high solubility, stable bulk properties, and consistent glass transition temperatures, indicating suitability for storage and handling. Phenolic and flavonoid contents varied among formulations, with S4 showing the highest phenolic concentration and sensory acceptability, while S1 had the highest flavonoid content and radical scavenging activity. FTIR analysis revealed vibrational signatures consistent with proteins, carbohydrates, sugars, and polyphenols, supported by quantitative assays demonstrating high retention of macronutrients and bioactives. This substantiates the particulates’ potential as functionally stable nutrient delivery systems. Future work should explore on the optimization of the formulation to further enhance sensory properties and antioxidant activity. Additionally, incorporating other functional ingredients and scaling up production processes could widen the scope for commercial applications. The particulates’ potential for use in functional food products, particularly as a convenient and nutrient‐rich supplement, deserves further investigation to maximize health benefits and consumer appeal.

## Disclosure

All authors read and approved the final manuscript.

## Conflicts of Interest

The authors declare no conflicts of interest.

## Author Contributions

**Pramod K. Prabhakar:** investigation, data curation, conceptualization, writing – original draft, resources. **Sanket Yadav:** investigation, data curation. **Yogesh Kumar:** formal analysis, data curation, writing – original draft. **Rajat Suhag:** formal analysis, data curation, conceptualization, methodology, software, writing – original draft, writing – review and editing. **Giovanna Ferrentino:** writing – review and editing.

## Funding

No funding was received for this manuscript.

## Data Availability

The data that support the findings of this study are available from the corresponding author upon reasonable request.
